# Development of novel surface display platforms for anchoring heterologous proteins in *Saccharomyces cerevisiae*

**DOI:** 10.1186/s12934-019-1133-x

**Published:** 2019-05-18

**Authors:** Xiaoyu Yang, Hongting Tang, Meihui Song, Yu Shen, Jin Hou, Xiaoming Bao

**Affiliations:** 1 State Key Laboratory of Microbial Technology, Shandong University, Qingdao, 266237 People’s Republic of China; 2 Shandong Provincial Key Laboratory of Microbial Engineering, Qi Lu University of Technology, Jinan, 250353 People’s Republic of China; 3 Center for Synthetic Biochemistry, Chinese Academy of Sciences, Shenzhen Institutes for Advanced Technologies, Shenzhen, 518055 People’s Republic of China

**Keywords:** Yeast surface display, Aga1, Dan4, Sed1, a-Agglutinin, Glycosylphosphatidylinositol (GPI)

## Abstract

**Background:**

Cell surface display of recombinant proteins has become a powerful tool for biotechnology and biomedical applications. As a model eukaryotic microorganism, *Saccharomyces cerevisiae* is an ideal candidate for surface display of heterologous proteins. However, the frequently used commercial yeast surface display system, the a-agglutinin anchor system, often results in low display efficiency.

**Results:**

We initially reconstructed the a-agglutinin system by replacing two anchor proteins with one anchor protein. By directly fusing the target protein to the N-terminus of Aga1p and inserting a flexible linker, the display efficiency almost doubled, and the activity of reporter protein α-galactosidase increased by 39%. We also developed new surface display systems. Six glycosylphosphatidylinositol (GPI) anchored cell wall proteins were selected to construct the display systems. Among them, Dan4p and Sed1p showed higher display efficiency than the a-agglutinin anchor system. Linkers were also inserted to eliminate the effects of GPI fusion on the activity of the target protein. We further used the newly developed Aga1p, Dan4p systems and Sed1p system to display exoglucanase and a relatively large protein β-glucosidase, and found that Aga1p and Dan4p were more suitable for immobilizing large proteins.

**Conclusion:**

Our study developed novel efficient yeast surface display systems, that will be attractive tools for biotechnological and biomedical applications

**Electronic supplementary material:**

The online version of this article (10.1186/s12934-019-1133-x) contains supplementary material, which is available to authorized users.

## Background

Cell surface display expresses a target protein or peptide on the cell surface through fusion of the target protein to an anchor protein. As a eukaryotic model microorganism, *Saccharomyces cerevisiae* (*S. cerevisiae*) is ideal for cell surface display because: (i) it is a “generally regarded as safe” microorganism; (ii) it is suitable for expressing eukaryotic proteins because post-translational modifications are conserved in eukaryotic organisms; (iii) its clear genetic background facilitates diverse genetic engineering; and (iv) its rapid growth is time-saving [1]. Yeast surface display systems have been used widely in vaccine and antibody development, library screening, biosensor detection systems, and bioconversion [2]. Yeast surface display has become a powerful protein engineering tool for applications in biotechnology and biomedicine [3, 4].

Generally, heterologous proteins are fused with the anchor domain of cell wall proteins for surface display [5–7]. In *S. cerevisiae*, the cell wall proteins are composed of two major classes—: (i) glycosylphosphatidylinositol (GPI) proteins, and (ii) the family of proteins with internal repeats (PIR) [8]. PIR proteins are attached to β-1,3-glucan directly in the cell wall, through an alkali-sensitive linkage [9]. In *S. cerevisiae*, the PIR family includes Pir1p, Pir2p, Pir3p, Pir4p and Pir5p. GPI proteins contain a GPI anchor which is synthesized in the endoplasmic reticulum and transferred to the carboxyl terminus of the protein. These proteins are then transported to the cell wall through the Golgi apparatus [10]. Nearly 60 GPI proteins have been identified in *S. cerevisiae* [8, 11].

The most frequently used yeast display system is the commercialized GPI protein a-agglutinin system. This system consists of two subunits, Aga1p and Aga2p. Heterologous proteins are fused to the C-terminus of Aga2p, Aga2p forms a complex with Aga1p via disulfide bonds, and Aga1p is anchored to the yeast surface through a GPI-anchor. The a-agglutinin system has been applied widely in vaccine and antibody development, library screening and biosensor detection systems. For example, a potential oral live vaccine against chicken coccidiosis was developed by displaying the *Eimeria tenella* EtMic2 protein on the cell surface via a-agglutinin [12]. A single-chain Fv (scFv) antibody library was anchored on the cell surface via a-agglutinin to screen scFvs that recognize various tumor marker antigens [13]. A bioreceptor mutation library was displayed on the cell surface to improve both the stability and the affinity toward ligands [14]. Although a-agglutinin is a widely used display system, the system requires two components that need to form a complex, i.e. Aga1p and Aga2p. In addition, the heterologous proteins anchored on the cell surface via disulfide bonds may not be stable under reducing condition. The two-component a-agglutinin system has been reported to have lower display efficiency than a one-component system, such as the α-agglutinin system [15]. Therefore, construction of new high-efficiency display systems for use in *S. cerevisiae* should be advantageous over available two-component system.

Several yeast surface display systems have been developed using GPI anchor proteins [16]. For example, the anchor domains of GPI proteins, including Cwp1p, Cwp2p, Aga1p, Tip1p, Flo1p, Sed1p, YCR89w, and Tir1p, were fused with α-galactosidase (α-Gal) and the display efficiency was compared. Cwp2p and Sed1p showed higher surface display levels than the other proteins [7]. When β-glucosidase (BGL1) and endoglucanase II (EGII) were displayed using the Sed1p anchoring region and *SED1* promoter, the enzyme activity was significantly higher than when using α-agglutinin (Sag1) system [17]. Although Sed1p-based display is highly efficient, using one anchor protein to display multiple proteins may lead to display saturation and relatively low display efficiency of each protein. Notably, disruption of *SED1* improved the display efficiency of BGL1 fused with Sed1p [18]. Therefore, developing new efficient yeast surface display systems remains essential for industrial applications.

In this study, we first reconstructed the a-agglutinin system, using only the GPI-containing domain of Aga1p as the anchor protein, to improve the display efficiency of heterologous proteins. Moreover, several new display systems were also developed, and among them, a Dan4p-based system showed the best display efficiency. Linkers were then added between heterologous proteins and anchor proteins to increase the activity of the heterologous proteins.

## Results

### Optimization of a-agglutinin anchor system to improve surface display efficiency

**Fig. 1:**
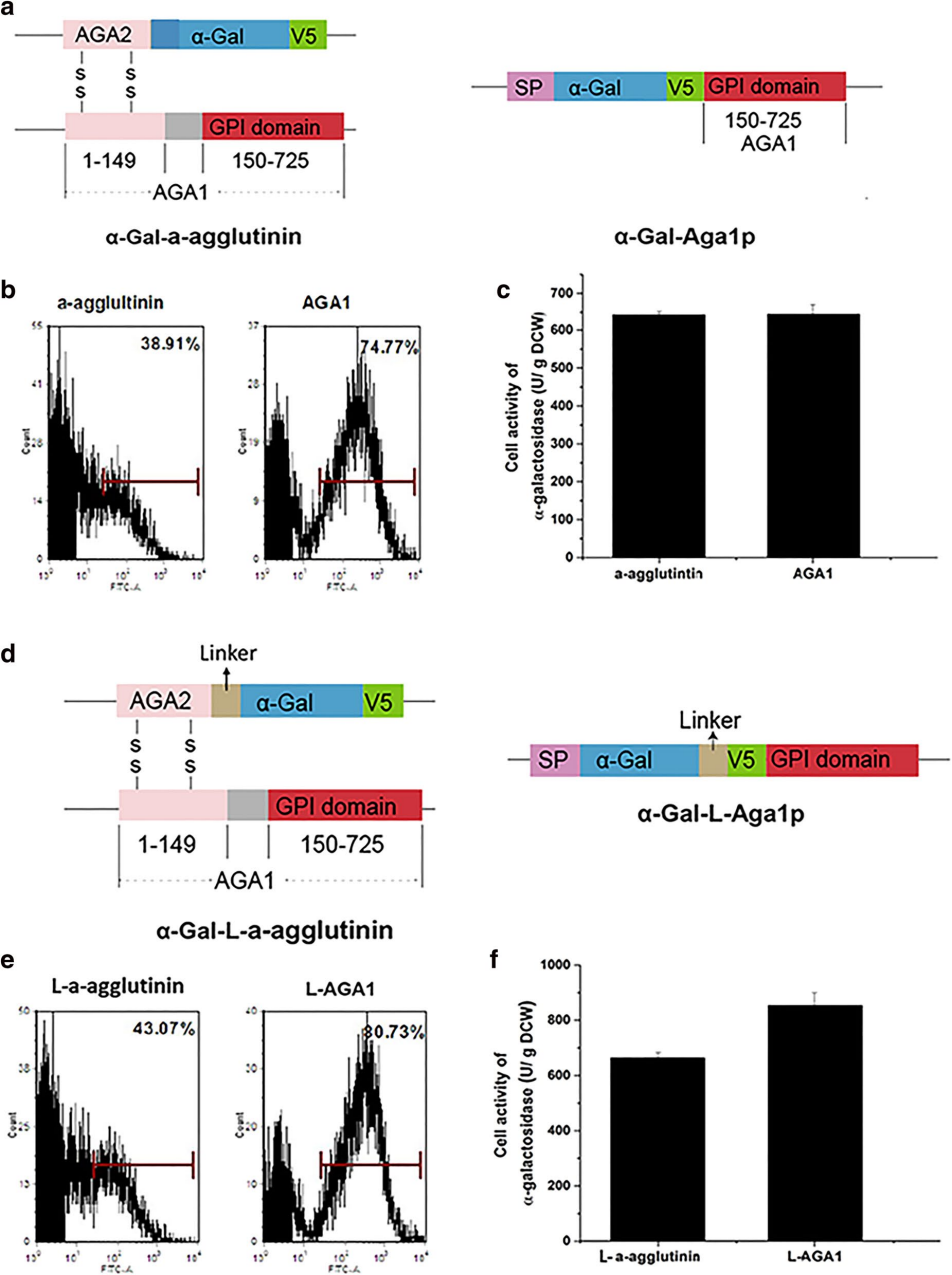
Optimization of the a-agglutinin anchor system. **a** Schematic representations of α-galactosidase (α-Gal)-a-agglutinin and α-Gal-Aga1p. **b** Flow cytometry analysis of α-Gal-a-agglutinin and α-Gal-Aga1p after 12-h incubation. **c** The α-Gal activities of α-Gal-a-agglutinin and α-Gal-Aga1p after 12-h incubation. **d** Schematic representation of α-Gal-Aga1p with a linker. **e** Flow cytometry analysis of α-Gal-a-agglutinin with a linker and α-Gal-Aga1p with a linker after 12-h incubation. **f** Cell activities of α-Gal-a-agglutinin with a linker and α-Gal-Aga1p linker after 12-h incubation

a-Agglutinin is a cell wall protein consisting of Aga1p and Aga2p. When used as a display system, a heterologous protein is normally fused to the C-terminus of Aga2p and co-expressed with Aga1p. The display of a heterologous protein depends on the formation of two disulfide bonds between Aga1p and Aga2p, which may result in low display efficiency. To improve the efficiency, we attempted to directly fuse the reporter protein α-Gal to the N-terminus of Aga1p (Fig. 1a). FACS analysis showed that the surface display efficiency of Aga1p was nearly doubled when compared with that for a-agglutinin (Fig. 1b). However, the cell activity of α-Gal-Aga1p was similar to that of α-Gal-a-agglutinin (Fig. 1c). This result indicated that the Aga1p based anchor system had higher display efficiency, but the activity of α-Gal did not increase as expected, presumably because the direct fusion of α-Gal with the N-terminus of Aga1p affected α-Gal activity. In a previous study, insertion of a linker consisting of Ser and Gly between anchor proteins and a lipase form *Rhizopus oryzae* improved the activity of the lipase [19]. Thus, a linker of 17 amino acids from the commercial plasmid pYD1 (Invitrogen, Carlsbad, California, USA) composed of Ser and Gly was inserted between Aga2p and α-Gal or α-Gal and Aga1p (Fig. 1d). After addition of the linker to both systems, the display efficiency of α-Gal-Aga1p was double that of α-Gal-a-agglutinin (Fig. 1e). The activity of α-Gal displayed by Aga1p increased 39% when compared with the activity of α-Gal-a-agglutinin (Fig. 1f). These results demonstrate that the one-protein-mediated Aga1p display system is better than the a-agglutinin display system for yeast cell surface display of heterologous protein.

### Development of a new surface display system

**Fig. 2:**
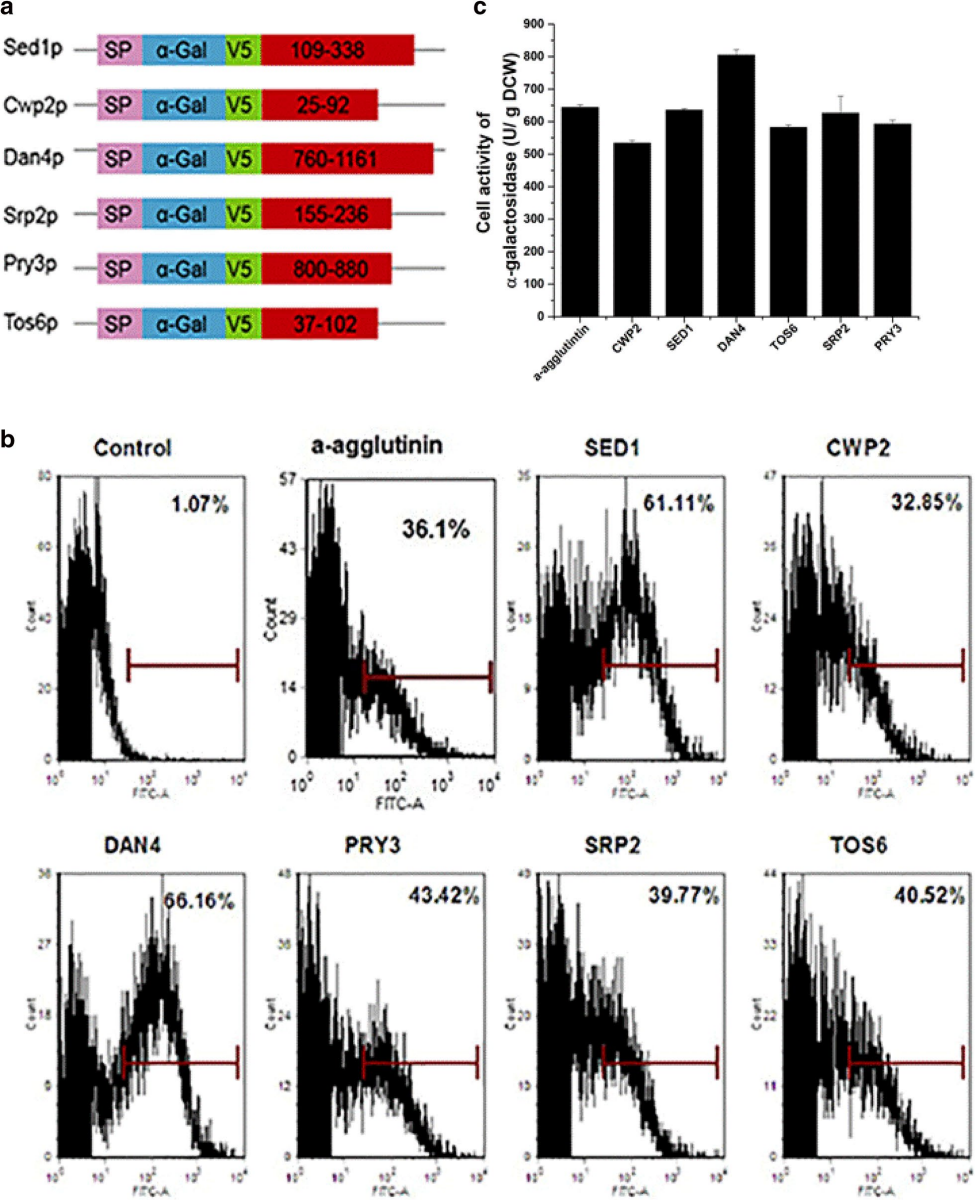
Comparison of immobilization efficiencies of different cell surface display systems. **a** Schematic representations of the surface display systems constructed in this study. **b** Flow cytometry analysis of different systems after 12-h incubation. **c** Cell enzyme activities of seven anchoring systems after 12-h incubation

To develop a new efficient yeast surface display system, we selected six proteins from putative GPI-dependent cell wall proteins according to previous reported study [20], and used their serine- and threonine-rich region and GPI domain for protein display. The proteins selected included two reported display proteins, Sed1p and Cwp2p, and four novel proteins, Dan4p, Tos6p, Srp2p and Pry3p. α-Gal was fused to the N-terminus of these selected anchor proteins. The design is shown in Fig. 2a. FACS analysis showed that α-Gal-Dan4p and α-Gal-Sed1p had higher display efficiency than α-Gal-a-agglutinin and the other anchor proteins (Fig. 2b). Interestingly, the trends of cell enzyme activities were not fully consistent with the display efficiency. α-Gal-Dan4p had the highest activity, and was 25% higher than that of α-Gal-a-agglutinin. The fusion of α-Gal with other anchor proteins did not increase enzyme activity (Fig. 2c). We observed that although α-Gal-Sed1p had high display efficiency, the fusion with Sed1p did not increase enzyme activity, indicating that the activity of α-Gal was affected by fusion with Sed1p.

**Fig. 3:**
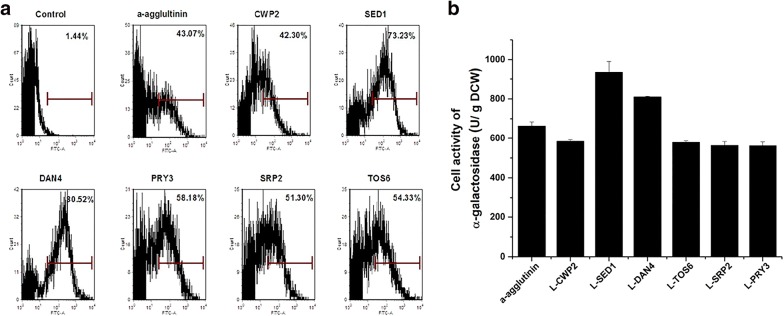
Comparison of immobilization efficiencies of cell surface display systems after inserting a linker peptide. **a** Flow cytometry analysis of different systems after 12-h incubation. **b** α-Gal activities of seven anchoring systems

In case there was an influence of the cell wall protein fusion on the target protein, we determined if enzyme activity could be enhanced by addition of a linker between the anchor protein and heterologous protein. The linker, containing 17 amino acids composed of Ser and Gly repeat sequence (as used above), was inserted between the anchor protein and the heterologous protein. After addition of the linker, the display efficiencies of these systems were improved by 6% to 14% (Fig. 3a). The enzyme activity was also enhanced by addition of the linker. α-Gal-Sed1p showed the greatest improvement (Fig. 3b) with the enzyme activity increasing by 40% when compared with that of α-Gal-Sed1p without the linker. However, for other anchor proteins, addition of the linker only improved α-Gal activity by 10% (Fig. 3b). The enzyme activity of α-Gal-Dan4p was not increased by linker addition.

Here we have identified a new efficient display system Dan4p based system. From the data above, we conclude that Aga1p, Dan4p and Sed1p are potential candidates for yeast cell surface display of heterologous proteins.

### Yeast surface display of two cellulases using the newly developed anchors

**Fig. 4:**
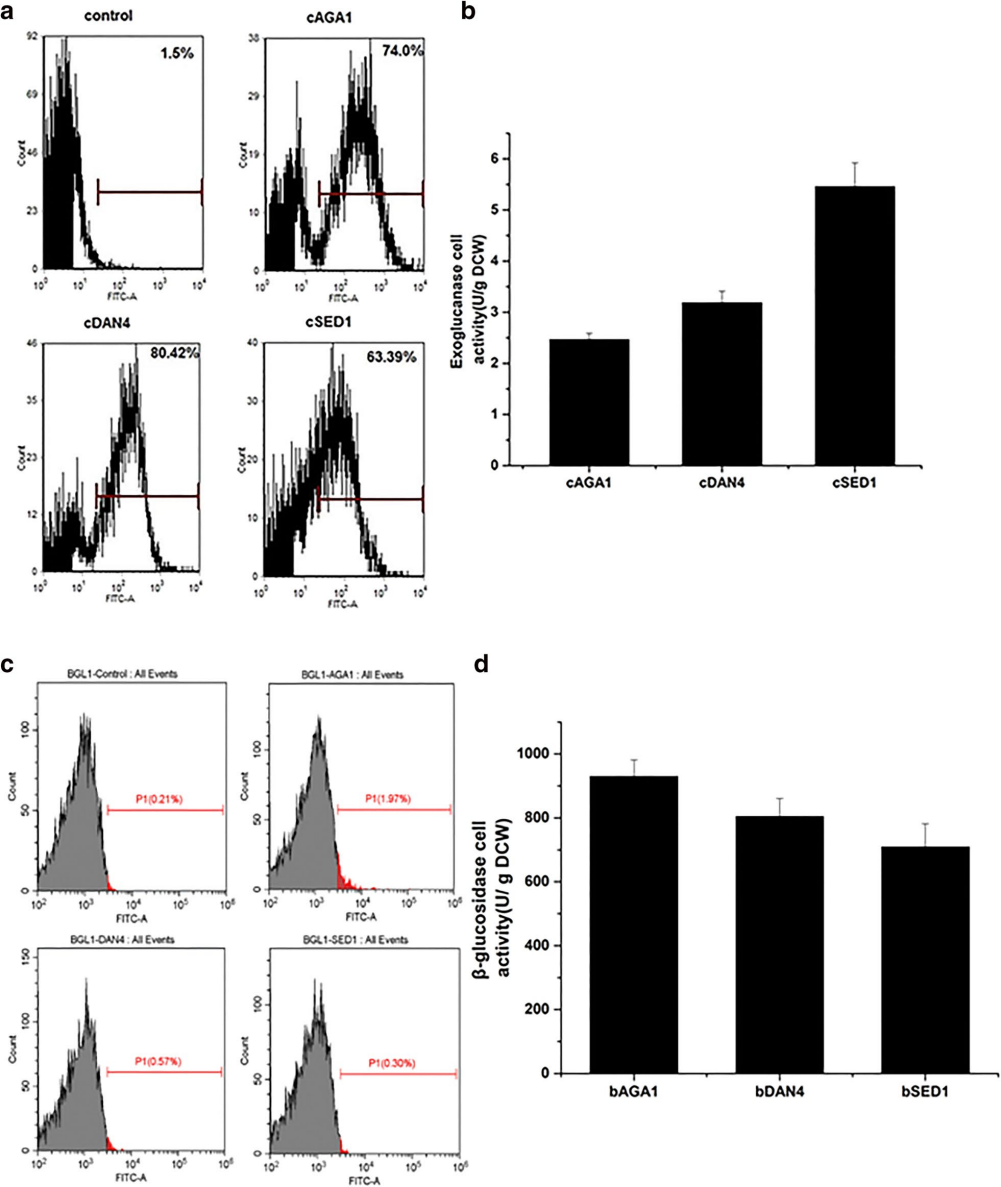
Comparison of cellulases displaying yeast strains. **a** Flow cytometry analysis of different CBH1-displaying systems after 12-h incubation. **b** Exoglucanase activities of CBH1-Aga1p, CBH1-Dan4p and CBH1-Sed1p. **c** Flow cytometry analysis of different BGL1-displaying systems after 12-h incubation. **d** β-glucosidase activities of BGL1-Aga1p, BGL1-Dan4p and BGL1-Sed1p

Exoglucanase (CBH1) and BGL1 are responsible for hydrolysis of cellulose to glucose, and play essential roles in cellulose degradation. Currently, display of cellulases, including CBH1 and BGL1, on the surface of yeast cells, are widely studied for the consolidated bioprocessing (CBP) of cellulosic ethanol production [3, 4]. Thus, to test the applicability of the new yeast surface display system, CBH1 from *Talaromyces emersonii* and BGL1 from *Saccharomycopsis fibuligera* were fused with Aga1p, Dan4p or Sed1p and displayed on the *Saccharomyces cerevisiae* cell surface. As shown in Fig. 4a, when displaying CBH1 on the cell surface, all three systems showed high display efficiencies, with the CBH1-Dan4p-containing strain yielding the highest display efficiency. The cell enzyme activity was not fully consistent with display efficiency. Among the three CBH1-containing strains, the CBH1-Sed1p-containing strain had the highest activity (Fig. 4b).

In a previous study, we found that positively stained cells of a BGL1-containing strain were not detected by FACS when using a-agglutinin as the display system [21]. FACS analysis showed that the display efficiency of BGL1-Aga1p- and BGL1-Dan4p-containing strains was higher than that of the BGL1-Sed1p-containing and control strain (Fig. 4c). However, the number of positive stained cells of the BGL1-Sed1p-containing strain was not much higher than that of the control strain. The cell activity results were consistent with the FACS results. The cell activities of BGL1-Aga1p and BGL1-Dan4p were also higher than that of BGL1-Sed1p (Fig. 4d). BGL1-Aga1p had the best performance among the systems examined. Compared with α-Gal (39.8 kDa) and CBH1 (48.7 kDa), BGL1 is a relatively large protein with a theoretical molecular weight of 96.2 kDa, and display on the cell wall may be more difficult. Thus, Aga1p and Dan4p may be more suitable for immobilizing large proteins.

## Discussion

Yeast surface display is a very useful tool for protein engineering, and has wide biotechnological and biomedical applications. It can be used for construction of whole-cell biocatalysts, protein engineering, protein library screening, identifying protein–protein interactions, and mapping protein epitopes [22–29]. For efficient cell surface engineering, it is important to choose an appropriate anchor protein to obtain high display efficiency. Several yeast surface display systems anchored by Sed1p, Aga1-Aga2, Agα1, and Spi1p have been reported [15, 17, 30–32]. With the increasing needs of biomedical and industrial applications, further efficient yeast surface display systems are required.

Herein, firstly, we reconstructed the commercialized a-agglutinin surface display system, which normally consists of two subunits (Aga1p and Aga2p). The new Aga1p system developed herein directly anchored the target protein on the cell surface, and did not require the Aga2p subunit, which is frequently fused with the N-terminus of the heterologous protein, and assembles with the cell wall-anchored Aga1p via disulfide bonds. Disulfide bonds are complicated and sensitive to redox conditions, and our new Aga1p system avoids the potential drawbacks to provide a more stable system. The new system also showed higher display efficiency when compared with that of the standard two-component system.

In addition, we selected different cell wall proteins to construct novel display systems. By comparing the display efficiencies and enzyme activities of several display systems, we found that Dan4p showed good performance in surface display efficiency, similar to that of Sed1p. Recently, Phienluphon et al. [33] used bioinformatic analysis to identify potential anchoring proteins, and found that Dan4p had higher display efficiency than Agα1p, which is consistent with our results. More importantly, the surface display efficiency of the Dan4p system was not affected by the addition of a linker, whereas the Sed1p system is dependent on the linker. Thus, three display systems, containing Aga1p, Dan4p and Sed1p were selected as potential systems for immobilizing recombinant proteins on the surface of yeast.

In a previous study, we found that even though the cellulolytic enzyme BGL1, a large protein, was displayed on the yeast cell surface by the Aga1p and Aga2p system, positive stained cells were not detected by FACS analysis, whereas display of the endoglucanases CelA on the cell surface was detected by FACS. We hypothesized that this is because of the large size of BGL1 and the relatively low display efficiency with a-agglutinin. Here, we used Sed1p, Aga1p and Dan4p to display BGL1 to further compare these systems. Interestingly, the BGL1-Aga1p and BGL1-Dan4p systems showed higher cell enzyme activities and display efficiency than BGL1-Sed1p. The BGL1-Aga1p system had more obvious advantage. Compared with Sed1p, Aga1p and Dan4p have a serine- and threonine-rich region and this region confers a rod-like structure to these two proteins [34]. This Ser/Thr rich region promotes cell wall integration and assists the cell wall protein to display their N-terminus outside the cell wall [11]. The serine- and threonine-rich region of Aga1p and Dan4p also increases the distance between the target protein and the anchor protein, thereby reducing potential spatial interference between anchor and displayed proteins. Therefore, Aga1p and Dan4p may be more suitable for displaying large proteins.

## Conclusions

Here, we reconstructed the a-agglutinin display system, and used one subunit (Aga1p) for enhancing the display efficiency of heterologous proteins. Moreover, several novel anchoring proteins were compared. Sed1p, Aga1p and Dan4p showed good performance, and are candidates for displaying heterologous proteins on the yeast cell wall. These new display systems will be attractive tools for biotechnological and biomedical applications.

## Methods

### Strains and media

*Escherichia coli* strain Trans5α (TransGen Biotech, Beijing, China) was used for plasmid propagation. Recombinant *E. coli* strains were grown in Luria–Bertani medium (5 g/L yeast extract, 10 g/L peptone and 20 g/L NaCl) with 100 mg/mL of ampicillin at 37 °C. *S. cerevisiae* strain CEN.PK102-5B [35] was used as the background strain for surface display of heterologous proteins and was cultivated in YPD medium (1% yeast extract, 2% peptone and 2% glucose). Recombinant strains were grown in SC-2×SCAA medium without uracil, at 30 °C, as previously described [36]. SC-2×SCAA is composed of 6.9 g/L yeast nitrogen base minus amino acids, 20 g/L glucose, 2 g/L KH_2_PO_4_ (pH 6 by KOH), 190 mg/L arginine, 52 mg/L tyrosine, 108 mg/L methionine, 290 mg/L isoleucine, 440 mg/L lysine, 200 mg/L phenylalanine, 400 mg/L aspartic acid, 1260 mg/L glutamic acid, 380 mg/L valine, 220 mg/L threonine, 400 mg/L leucine, 130 mg/L glycine, 40 mg/L tryptophan and 140 mg/L histidine.

### Plasmid construction

Genes for heterologous expression and primers were synthesized and recombinant genes were sequenced by GENEWIZ (Suzhou, China). Plasmids and primers used are listed in Additional file 1: Tables S1 and S2, respectively. The α-Gal gene [20] with signal peptide *SUC2* from *S. cerevisiae* and a V5 tag or V5 tag with linker were synthesized and ligated into the yeast plasmid pJFE3 [37] under the control of the *TEF1* promoter and *PGK1* terminator to construct plasmids pJαg and pJαg-Linker. The C-terminal sequences of *SED1* (687 bp), *CWP2* (201 bp), *SRP2* (243 bp), *DAN4* (1203 bp), *PRY3* (240 bp), *TOS6* (195 bp) and *AGA1* (1629 bp), for use in surface display systems, were amplified from *S. cerevisiae* genomic DNA, and were ligated into pJαg and pJαg-Linker using Gibson assembly [38]. For surface display via a-agglutinin, the α-Gal gene fused with *AGA2* was ligated into pJFE3, and *AGA1* (full-length) was also cloned into the plasmid to construct pJαg-AGA12. The *CBH1* gene was synthesized and ligated into pJαg-L-AGA1, pJαg-L-DAN4 and pJαg-L-SED1 to construct pJCBH-L-AGA1, pJCBH-L-DAN4 and pJCBH-L-SED1 respectively. The *BGL1* gene was amplified from the plasmid pTH-BGL [21] and ligated into pJαg-L-AGA1, pJαg-L-DAN4 and pJαg-L-SED1 to construct pJBGL-L-AGA1, pJBGL-L-DAN4 and pJBGL-L-SED1 respectively.

### Enzyme assays

α-Gal activity was measured using *p*-nitrophenyl-α-d-galactopyranoside (*p*NPGal; Sigma-Aldrich, St Louis, MO, USA) as the substrate as described previously [20]. Cells were collected and washed twice with 100 mM sodium acetate buffer (pH 4.5). The cell activity of α-Gal was then measured. The cells were suspended in 100 mM sodium acetate (pH 4.5) and incubated in 50 mM citrate buffer (pH 4.5) with 5 mM *p*NPGal for 5 min at 37 °C. The reaction was stopped by the addition of 1 mL of 2% (wt/vol) sodium carbonate and *p*-nitrophenol (*p*NP) released from *p*NPGal was determined at 405 nm. One enzyme unit is defined as the amount of enzyme required to release 1 μmol of *p*NP per min in the assay conditions.

CBH1 activity was measured using *p*-nitrophenyl-β-d-cellobioside (*p*NPC) as the substrate (Sigma) [39]. Cells were washed with 50 mM citrate buffer (pH 4.8) and then incubated in 50 mM citrate buffer (pH 4.8) with 5 mM *p*NPC for 30 min at 50 °C. The reaction was terminated by the addition of 150 μL 10% (wt/vol) sodium carbonate and the *p*-nitrophenol (*p*NP) released from *p*NPC was determined at 405 nm. One enzyme unit is defined as the amount of enzyme required to release 1 μmol of *p*NP per minute under the assay conditions.

BGL1 activity was measured using *p*-nitrophenyl-β-d-glucopyranoside (*p*NPG; Sigma) as the substrate, as described previously [40]. Cells were washed with 50 mM citrate buffer (pH 5.0) and then incubated with 5 mM *p*NPG for 30 min at 50 °C. The reaction was terminated by addition of 10% (wt/vol) sodium carbonate and *p*-nitrophenol (*p*NP) released from *p*NPG was determined at 405 nm. One enzyme unit is defined as the amount of enzyme required to release 1 μmol of *p*NP per min under the assay conditions.

### Flow cytometry analysis

Cells were harvested and washed twice with phosphate buffered saline (PBS, pH 7.0), and suspended in PBS with 1 mg/mL bovine serum albumin (BSA). The cells were diluted to an OD_600_ of 1.0 and 300 μL of cell suspension was used as the sample for immunostaining. The monoclonal mouse anti-V5-FITC antibody (Invitrogen) was added to the samples at 1: 500 dilution at 25 °C for 1 h. The cells were harvested and washed twice with PBS after immunostaining. Flow cytometry analysis (FACS) used the FACSCanto II system (BD FACSCanto II, USA) and the CytoFLEX Platform (BECKMAN COULTER, USA).

## Additional file

**Additional file 1: Table S1.** Plasmids used in this study. **Table S2.** Primer sequences used in this study.

## Data Availability

All data generated or analyzed during this study are included in this published article and its Additional files.
