# Supplementary material for: Development of novel surface display platforms for anchoring heterologous proteins in Saccharomyces cerevisiae
Source: Microb Cell Fact. 2019 May 18;18:85. doi: 10.1186/s12934-019-1133-x (PMC6525377; doi:10.1186/s12934-019-1133-x)
Supplement: Supplementary file 1 — Additional file 1: Table S1. Plasmids used in this study. Table S2. Primer sequences used in this study. [file 12934_2019_1133_MOESM1_ESM.docx]

**Development of novel surface display platforms for anchoring heterologous proteins in *Saccharomyces cerevisiae***

Xiaoyu Yang^1#^, Hongting Tang^1,3#^, Meihui Song^1^, Yu Shen^1^, Jin Hou^1^*, Xiaoming Bao^1,2^*

^1^ State Key Laboratory of Microbial Technology, Shandong University, Qingdao, 266237, P. R. China

^2^Shandong Provincial Key Laboratory of Microbial Engineering, Qi Lu University of Technology, Jinan, 250353, P. R. China

^3^Center for Synthetic Biochemistry, Chinese Academy of Sciences, Shenzhen Institutes for Advanced Technologies, Shenzhen, 518055, P. R. China

^#^ To be considered as joint first authors.

* Corresponding author: Prof. Xiaoming Bao, email: [bxm@sdu.edu.cn](mailto:bxm@sdu.edu.cn); Prof. Jin Hou, email: [houjin@sdu.edu.cn](mailto:houjin@sdu.edu.cn), State Key Laboratory of Microbial Technology, Shandong University, Qingdao, 266237, P. R. China, Tel/Fax: +86 532 58632401

**Table S1.** Plasmids used in this study.

| **Plasmid** | **Description** | **Source** |
| --- | --- | --- |
| pJFE3 | 2µm, amp^r^, *TEF1p-PGK1t, URA3* | Lab store |
| pJαg | 2µm, amp^r^, *TEF1*p-*SUC2*-*α-gal-V5*-*PGK1*t, *URA3* | This study |
| pJαg-AGA12 | 2µm, amp^r^, *TEF1*p-*AGA1* -*PGK1*t, *TEF1*p-*AGA*2-*α-gal-V5* -*PGK1*t*, URA3* | This study |
| pJαg-AGA1 | 2µm, amp^r^, *TEF1*p-*SUC2*-*α-gal-V5*-*AGA1*-*PGK1*t, *URA3* | This study |
| pJαg-CWP2 | 2µm, amp^r^, *TEF1*p-*SUC2-α-gal-V5*-*CWP2*-*PGK1*t, *URA3* | This study |
| pJαg-DAN4 | 2µm, amp^r^, *TEF1*p-*SUC2-α-gal-V5*-*DAN4*-*PGK1*t, *URA3* | This study |
| pJαg-PRY3 | 2µm, amp^r^, *TEF1*p-*SUC2-α-gal-V5*-*PRY3*-*PGK1*t, *URA3* | This study |
| pJαg-SED1 | 2µm, amp^r^, *TEF1*p-*SUC2-α-gal-V5*-*SED1*-*PGK1*t, *URA3* | This study |
| pJαg-SRP2 | 2µm, amp^r^, *TEF1*p-*SUC2-α-gal-V5*-*SRP2*-*PGK1*t, *URA3* | This study |
| pJαg-TOS6 | 2µm, amp^r^, *TEF1*p-*SUC2-α-gal-V5*-*TOS6*-*PGK1*t, *URA3* | This study |
| pJαg-Linker | 2µm, amp^r^, *TEF1*p- *V5-*linker-*PGK1*t, *URA3* | This study |
| pJαg-L-AGA12 | 2µm, amp^r^, *TEF1*p-*AGA1* -*PGK1*t, *TEF1*p-*AGA*2-linker*-V5-α-gal*-*PGK1*t, *URA3* | This study |
| pJαg-L-AGA1 | 2µm, amp^r^, *TEF1*p-*SUC2-α-gal-*linker*-V5*-*AGA1*-*PGK1*t, *URA3* | This study |
| pJαg-L-CWP2 | 2µm, amp^r^, *TEF1*p-*SUC2-α-gal-*linker*-V5*-*CWP2*-*PGK1*t, *URA3* | This study |
| pJαg-L-DAN4 | 2µm, amp^r^, *TEF1*p-*SUC2-α-gal-*linker*-V5*-*DAN4*-*PGK1*t, *URA3* | This study |
| pJαg-L-PRY3 | 2µm, amp^r^, *TEF1*p-*SUC2-α-gal-*linker*-V5*-*PRY3*-*PGK1*t, *URA3* | This study |
| pJαg-L-SED1 | 2µm, amp^r^, *TEF1*p-*SUC2-α-gal-*linker*-V5*-*SED1*-*PGK1*t, *URA3* | This study |
| pJαg-L-SRP2 | 2µm, amp^r^, *TEF1*p-*SUC2-α-gal-*linker*-V5*-*SRP2*-*PGK1*t, *URA3* | This study |
| pJαg-L-TOS6 | 2µm, amp^r^, *TEF1*p-*SUC2-α-gal-*linker*-V5*-*TOS6*-*PGK1*t, *URA3* | This study |
| pJCBH-L-AGA1 | 2µm, amp^r^, *TEF1*p-*CBH1-*linker*-V5*-*AGA1*-*PGK1*t, *URA3* | This study |
| pJCBH-L-DAN4 | 2µm, amp^r^, *TEF1*p-*CBH1-*linker*-V5*-*DAN4*-*PGK1*t, *URA3* | This study |
| pJCBH-L-SED1 | 2µm, amp^r^, *TEF1*p-*CBH1-*linker*-V5*-*SED1*-*PGK1*t, *URA3* | This study |
| pJBGL-L-AGA1 | 2µm, amp^r^, *TEF1*p-*BGL-*linker*-V5*-*AGA1*-*PGK1*t, *URA3* | This study |
| pJBGL-L-DAN4 | 2µm, amp^r^, *TEF1*p-*BGL-*linker*-V5*-*DAN4*-*PGK1*t, *URA3* | This study |
| pJBGL-L-SED1 | 2µm, amp^r^, *TEF1*p-*BGL-*linker*-V5*-*SED1*-*PGK1*t, *URA3* | This study |

**Table S2.** Primer sequences used in this study.

| Name | Sequence | purpose |
| --- | --- | --- |
| AGA2-F | CTAATCTAAGTTTTAATTACAAAGGATCCTCTAGAATGCAGTTACTTCGCTGTTTTTC | Obtain *AGA2* full length, construct pJαg-AGA12 |
| AGA2-R | CATTGGTGGAGTCTGACCCAAACCGTTTTCAGCAAAAACATACTGTGTGTTTATGGGGC |  |
| TEFp-AGA1-F | GTCACGACGTTGTAAAACGACGGCCAGTGAATTCCCACACACCATAGCTTCAAAATG | Obtain *AGA1* full length, construct pJαg-AGA12 |
| TEFp-R | ATAATGTCATTCTAGAGGATCCTTTGTAATTAAAACTTAGATTAGATTGCTATGCTTTC |  |
| AGA1-TEFp-F | TAAGTTTTAATTACAAAGGATCCTCTAGAATGACATTATCTTTCGCTCAT |  |
| AGA1-PGKt-R | CAATCCTGCAGGTCGACTCTAGATTAACTGAAAATTACATTGCAAGCAAC |  |
| PGKt-F | ATTTTCAGTTAATCTAGAGTCGACCTGCAGGATTGAATTGAATTGAAATCGATAGATC |  |
| PGKt- AGA1 -R | ACGTACAAAGTATGCATTGTGGTACCGAGCTCGAATTCAACGCAGAATTTTCGAGTTAT |  |
| AGA1-F | CCTTTGTTGGGTTTAGATTCTACTGTCGACCTGCAGACTACCTCCCTAAGTTCGAC | Obtain C-terminal sequences of *AGA1* |
| AGA1-R | TATCGATTTCAATTCAATTCAATCCTGCAGGTCGAACTGAAAATTACATTGCAAGC |  |
| CWP2-F | TCCTTTGTTGGGTTTAGATTCTACTGTCGACCTGCAGATTTCTCAAATCACTGACG | Obtain C-terminal sequences of *CWP2* |
| CWP2-R | GATCTATCGATTTCAATTCAATTCAATCCTGCAGGTTATAACAACATAGCAGCAGCAG |  |
| DAN4-F | TGTTGGGTTTAGATTCTACTGCATGCACTAGTGTCGACCTGCAGTCAGTCGCATCTTTTGCATC | Obtain C-terminal sequences of *DAN4* |
| DAN4-R | CTATCGATTTCAATTCAATTCAATCCTGCAGGTTATAGCAGTAATAAAGCGACAACAG |  |
| PRY3-F | TCCTTTGTTGGGTTTAGATTCTACTGTCGACCTGCAGTCCAGCACAAGCCTAGGTG | Obtain C-terminal sequences of *PRY3* |
| PRY3-R | TGATCTATCGATTTCAATTCAATTCAATCCTGCAGGTTAGGCGAACAGAACAGCTAC |  |
| SED1-F | TCCTTTGTTGGGTTTAGATTCTACTGTCGACCTGCAGGCTCTTCCAACTAACGGTAC | Obtain C-terminal sequences of *SED1* |
| SED1-R | CAATTCAATTCAATCCTGCAGGTCGATAAGAATAACATAGCAACACCAGCCAAAC |  |
| SRP2-F | CTTTGTTGGGTTTAGATTCTACTGTCGACCTGCAGATGAGCATCATCAATAATAGTG | Obtain C-terminal sequences of *SRP2* |
| SRP2-R | CTATCGATTTCAATTCAATTCAATCCTGCAGGTTAAGGGTGAAGTAACAAGTACAG |  |
| TOS6-F | CTTTGTTGGGTTTAGATTCTACTGTCGACCTGCAGACATCTATGGTCTCCACCG | Obtain C-terminal sequences of *TOS6* |
| TOS6-R | GATCTATCGATTTCAATTCAATTCAATCCTGCAGGTTATAACAAAGCAAAGGCAGCAG |  |
| AGA1-L-F | GTGGTGGTTCTGCATGCACTAGTGTCGACCTGCAGACTACCTCCCTAAGTTCGAC | Construct plasmids pJαg-L-AGA1 |
| AGA1-L-R | CTATCGATTTCAATTCAATTCAATCCTGCAGGTTAACTGAAAATTACATTGCAAGC |  |
| CWP2-L-F | TGGTGGTGGTTCTGCATGCACTAGTGTCGACCTGCAGATTTCTCAAATCACTGACGGTC | Construct plasmids pJαg-L-DAN4 |
| CWP2-L -R | GATCTATCGATTTCAATTCAATTCAATCCTGCAGGTTATAACAACATAGCAGCAGCAG |  |
| DAN4-L-F | GGTGGTTCTGCATGCACTAGTGTCGACCTGCAGTCAGTCGCATCTTTTGCATC | Construct plasmids pJαg-L-CWP2 |
| DAN4-L -R | CTATCGATTTCAATTCAATTCAATCCTGCAGGTTATAGCAGTAATAAAGCGACAACAG |  |
| PRY3-L-F | GGTGGTGGTGGTTCTGCATGCACTAGTGTCGACCTGCAGTCCAGCACAAGCCTAGGTG | Construct plasmids pJαg-L-PRY3 |
| PRY3-L-R | TGATCTATCGATTTCAATTCAATTCAATCCTGCAGGTTAGGCGAACAGAACAGCTAC |  |
| SED1-L-F | GGTGGTTCTGCATGCACTAGTGTCGACCTGCAGGCTCTTCCAACTAACGGTACTTCTA | Construct plasmids pJαg-L-SED1 |
| SED1-L -R | TCGATTTCAATTCAATTCAATCCTGCAGGTTATAAGAATAACATAGCAACACCAGC |  |
| TOS6-L-F | TGGTGGTGGTGGTTCTGCATGCACTAGTGTCGACCTGCAGACATCTATGGTCTCCACCG | Construct plasmids pJαg-L-TOS6 |
| TOS6-L-R | GATCTATCGATTTCAATTCAATTCAATCCTGCAGGTTATAACAAAGCAAAGGCAGCAG |  |
| CBH -F | CAATCTAATCTAAGTTTTAATTACAAAGGATCCATGTTAAGAAGAGCTTTGTTATTGT | Obtain *CBH1* full length |
| CBH-R | TAAACCCAACAAAGGATTTGGAATTGGCTTACCCTCGAGTGAAGCTGTAAATGTAGAGT |  |
| BGL -F | TCTAATCTAAGTTTTAATTACAAAGGATCCCATGTTGATGATAGTACAGCTTTTGGT | Obtain *BGL1* full length |
| BGL-R | CAAAGGATTTGGAATTGGCTTACCCTCGAGAATAGTAAACAGGACAGATGTCTTG |  |
